# Whole-Genome Analysis and Growth-Promoting Mechanism of *Klebsiella pneumoniae* YMK25 from Maize Rhizobacteria

**DOI:** 10.3390/plants14172738

**Published:** 2025-09-02

**Authors:** Xinhui Yu, Jinnan Xia, Shaojie Bi, Haipeng Wang, Changjiang Zhao

**Affiliations:** 1 College of Agriculture, Heilongjiang Bayi Agricultural University, Daqing 163319, China; 13588241571@163.com; 2 Heilongjiang Provincial Key Laboratory of Environmental Microbiology and Recycling of Argo-Waste in Cold Region, College of Life Science and Biotechnology, Heilongjiang Bayi Agricultural University, Daqing 163319, China; 18346502715@163.com (J.X.); bishaojie@byau.edu.cn (S.B.); 3 Key Laboratory of Low-Carbon Green Agriculture in Northeastern China, Ministry of Agriculture and Rural Affairs P. R. China, Heilongjiang Bayi Agricultural University, Daqing 163319, China; 4 School of Environment, Harbin Institute of Technology, Harbin 150090, China; 21b329007@stu.hit.edu.cn

**Keywords:** *Klebsiella pneumoniae*, plant growth-promoting rhizobacteria, maize, whole genome analysis, soil nutrient

## Abstract

Plant growth-promoting rhizobacteria (PGPR) are microorganisms that enhance plant growth through various mechanisms. In the context of global agriculture, which faces fertilizer dependency and environmental pollution, developing eco-friendly microbial fertilizers has become crucial for enhancing agricultural sustainability. To identify highly effective PGPR, we isolated 102 bacterial strains from maize rhizosphere soil using the dilution plating method. The strains were screened for growth-promoting abilities using functional media, resulting in the selection of strain YMK25 for its exceptional capabilities in nitrogen fixation, solubilization of inorganic and organic phosphorus, indole-3-acetic acid (IAA) production, and siderophore production. Strain YMK25 produced IAA at a concentration of 80.49 ± 0.68 μg/mL and exhibited a relative siderophore expression level of 43.68%. Morphological analysis, 16S rDNA gene sequence analysis, and whole-genome sequencing confirmed that strain YMK25 is *Klebsiella pneumoniae*. Whole-genome analysis revealed a total genome length of 5,115,280 bp, a GC content of 57.61%, and it contained 4746 coding genes. Gene annotation results indicated genes involved in siderophore synthesis, phosphatase activity, and other plant growth-promoting functions, which align with the verified characteristics of strain YMK25. Furthermore, this strain exhibited significant metabolic capabilities. The pot experiment demonstrated that strain YMK25 promotes maize plant growth and assists in nutrient fixation in these plants. In conclusion, strain YMK25 is a high-quality PGPR with substantial potential for application in agricultural production, presenting promise for widespread use in sustainable agriculture.

## 1. Introduction

Traditional agricultural practices that rely on fertilizers and pesticides to boost crop yields have resulted in soil degradation, environmental pollution, and ecological imbalances [1]. In this context, research on plant growth-promoting rhizobacteria (PGPR) has garnered attention as a strategy for adopting sustainable production methods and promoting green agriculture. These microorganisms enhance plant growth through various mechanisms, such as nitrogen fixation, solubilization of phosphorus and potassium, secretion of plant hormones, and improvement in soil structure [2]. They not only improve nutrient uptake by plants but also increase their resistance to stress, thereby enhancing crop growth and yield [3]. For instance, PGPR can convert nitrogen and phosphorus compounds in the soil into forms readily absorbed by plants [4]. Some PGPR strains can synthesize indole-3-acetic acid (IAA), a significant phytohormone that promotes root development and enhances plant stress tolerance [5]. Furthermore, PGPR can inhibit the growth of plant pathogens, thereby enhancing plant disease resistance [6]. Consequently, utilizing PGPR as a biofertilizer can reduce reliance on chemical fertilizers while enhancing crop productivity and improving plant resistance to various stresses.

Maize (*Zea mays* L.) is the principal staple crop in our country, with an annual planting area exceeding 20 million hectares [7]. To address the increasing competition for limited arable land resources and the rising demand for food and feed amid economic development, it is crucial to significantly enhance maize yield [8]. The microbial communities in the rhizosphere soil of maize play a crucial role in influencing its growth and development [9]. Researchers have identified several PGPR strains with excellent growth-promoting characteristics through the screening and isolation of rhizosphere microorganisms, which effectively improve the soil environment and promote plant growth [10]. For example, Wan et al. [11] isolated phosphate-solubilizing bacteria P24 and P43 from the rhizosphere soil of maize. These bacteria significantly promote the growth of maize seedlings, enhancing plant height, fresh weight, and root length. Jha et al. [12] studies indicate that the genome of *Klebsiella pneumoniae* PVN-1 has many advantageous traits that can be utilized to enhance plant growth and aid in phytoremediation. Liu et al. [13] found *Klebsiella pneumoniae* SnebYK can promote root development and increase the fresh weight of soybean seedlings. Liang et al. [14] indicate that *Klebsiella pneumoniae* can promote the growth of corn seedlings.

Whole genome sequencing allows for the sequencing and analysis of an organism’s entire genome. Analyzing the sequences and annotating functional genes enables a deeper understanding of the phenotypic mechanisms of the strains [15,16]. This study isolated a strain of *Klebsiella pneumoniae* YMK25 from the rhizosphere soil of maize and verified its growth-promoting function. To ensure the safety of the tested strain, a safety evaluation of strain YMK25 was conducted prior to the experiment in accordance with the NY/T 1109-2017 standard [17]. The results indicated that strain YMK25 is non-pathogenic [18]. Functional annotation and prediction of growth-promoting genes were carried out using whole-genome sequencing technology. By combining growth-promoting indicators, the research examined the strain’s effects on maize growth, with the goal of providing quality microbial resources for the development and application of microbial fertilizers for maize.

## 2. Results

A total of 102 strains were isolated from the maize rhizosphere soil. Among these strains, one strain designated as YMK25 was selected due to its exceptional abilities in nitrogen fixation, solubilization of inorganic and organic phosphorus, siderophore production, and IAA synthesis.

### 2.1. YMK25 Exhibits Superior Nitrogen Fixation and Phosphate Solubilization

**Figure 1:**
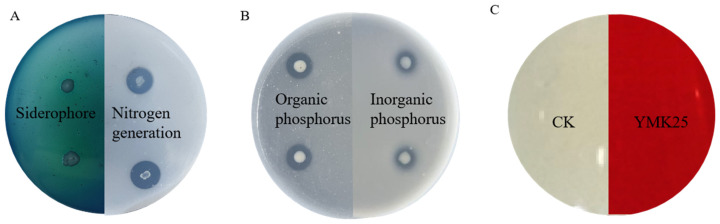
Colony characteristics related to growth enhancement capability. Note: (**A**): qualitative results of siderophore production and nitrogen fixation; (**B**): qualitative results of organic phosphorus and inorganic phosphorus; (**C**): IAA colorimetric reaction.

The nitrogen-fixing ability of strain YMK25 was qualitatively assessed using the Ashby medium. The strain exhibited robust growth, characterized by a viscous, semi-transparent appearance and a distinct clear zone (Figure 1A). The phosphate solubilization ability of strain YMK25 was qualitatively assessed using NBRIP agar medium and PSM agar medium, yielding solubility indices of 1.74 and 2.78, respectively (Figure 1B). Quantitative analysis revealed that strain YMK25 can solubilize 42.37 ± 1.28 mg/L of inorganic phosphorus and 73.25 ± 2.31 mg/L of organic phosphorus. These results confirm that YMK25 had a strong ability to dissolve phosphorus and fix nitrogen.

### 2.2. YMK25 Produced Siderophores at Excellent Expression Level

An assessment of strain YMK25’s ability to secrete siderophores on CAS medium revealed a distinct orange-yellow halo surrounding the strain (Figure 1A). Quantitative results indicated that the siderophore content exhibited a relative expression level of 43.68%, demonstrating that strain YMK25 is capable of producing siderophores.

### 2.3. YMK25 Possesses a Robust IAA Production Capacity

The IAA production capacity of strain YMK25 was preliminarily assessed using colorimetric methods. A red color appeared in the supernatant culture medium without L-tryptophan, confirming its ability to produce IAA (Figure 1C). Quantitative analysis revealed that strain YMK25 produces IAA at a concentration of 80.49 ± 0.68 mg/L.

### 2.4. Characterization of the Fundamental Physicochemical Properties of YMK25

**Figure 2:**
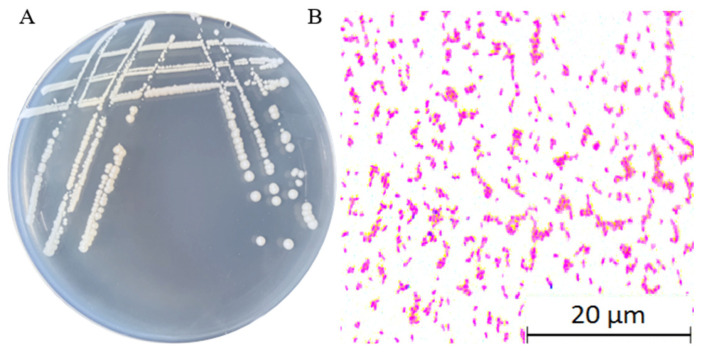
Colony morphology of strain YMK25. Note: (**A**) shows the morphology of strain YMK25 taken under normal lighting conditions, and (**B**) shows the Gram staining microscopic observation of strain YMK25 (10 × 100).

After 48 h of cultivation on LB medium, the colonies of strain YMK25 appeared milky white and translucent, featuring a smooth, raised, and moist surface with a neat, viscous edge (Figure 2A). Gram staining revealed that strain YMK25 is a Gram-negative bacterium, exhibiting a short rod shape and predominantly occurring as single cells (Figure 2B). The methyl red test, hydrogen sulfide production test, and gelatin hydrolysis test yielded negative results for strain YMK25, while the Voges–Proskauer (VP) test yielded a positive result. This strain can ferment both lactose and glucose. Additionally, it can utilize citrate, malonate, lysine decarboxylase, and urease; however, it cannot utilize arginine dihydrolase and ornithine. The physiological and biochemical characteristics of the strains are presented in Table 1.

### 2.5. Phylogenetic and Genomic Similarity Analysis

**Figure 3:**
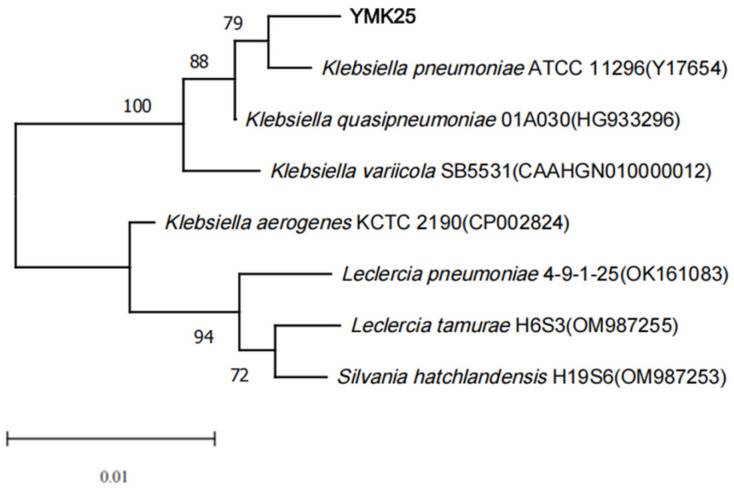
Phylogenetic tree based on 16S rDNA sequences.

The 16S rRNA gene sequence of strain YMK25 was 1442 bp in length. The phylogenetic tree constructed from the 16S rRNA gene sequence (Figure 3) indicates that strain YMK25 clustered at the same node as *Klebsiella pneumoniae* ATCC11296, demonstrating a 99% sequence identity. This indicates that strain YMK25 is closely related to *Klebsiella* spp.

Based on the results of the 16S rRNA gene sequence alignment, we selected model strains of *Klebsiella* spp that exhibit high similarity to the sequence of strain YMK25 for whole-genome comparison. The selected strains include *Klebsiella pneumoniae* ATCC 13883, *Klebsiella quasipneumoniae* 01A030, *Klebsiella variicola* SB 5531, and *Klebsiella aerogenes* KCTC 2109. Their genomic profiles are presented in Table 2. The tRNA and GC content ratio of YMK25 is closest to that of *Klebsiella pneumoniae* ATCC 13883, whereas the total genome length and the number of coding sequences (CDSs) are most similar to those of *Klebsiella aerogenes* KCTC 2109. To further understand the diversity of the strain’s genomes, a Core/Pan analysis was conducted, which identified a total of 24,212 homologous genes. These gene clusters were further categorized into the core genome, comprising 3445 homologous gene clusters that are universally present in all strains, and 2203 specific homologous gene clusters, among which strain YMK25 contains 185 specific homologous genes.

**Figure 4:**
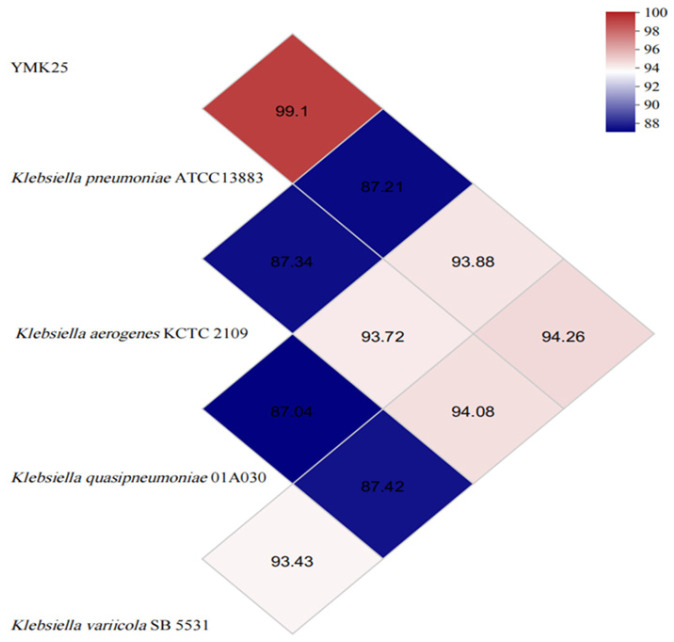
The ANI value based on whole-genome comparisons.

To classify strain YMK25 more accurately, ANI values between the genomes of five strains were calculated using whole-genome comparisons (Figure 4). The results showed that the maximum ANIm value between YMK25 and *Klebsiella pneumoniae* ATCC 13883 was 99.1%, which does not meet the criteria for species delineation. The Genome-to-Genome Distance Calculator 3.0 was used to calculate the DDH between strain YMK25 and *Klebsiella pneumoniae* ATCC11296, yielding a reference result of 87.60%. Based on these results, it can be concluded that strain YMK25 is classified as *Klebsiella pneumoniae*.

### 2.6. Whole Genome Characterization and Annotation of Strain YMK25

**Figure 5:**
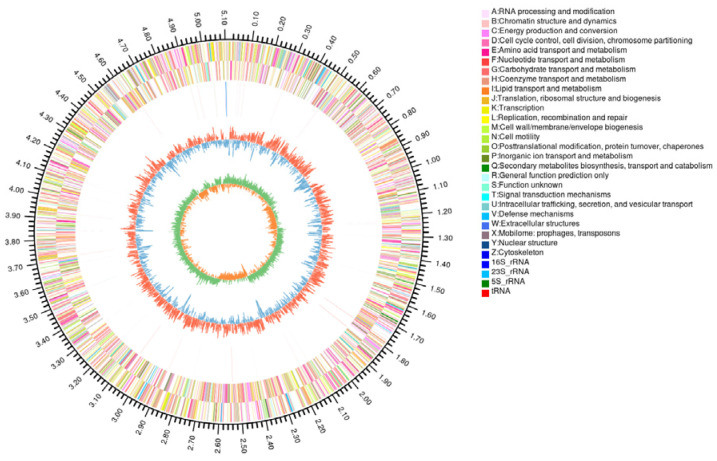
Circular map of the genome of strain YMK25.

Whole genome analysis of strain YMK25 revealed an assembled genome size of 5,115,280 bp and a GC content of 57.61%. GeneMarkS annotation predicted 4746 protein-coding genes, in addition to 79 tRNA, 6 rRNA, and 121 sRNA non-coding RNAs. The total length of the coding genes amounted to 4,518,210 bp, with an average gene length of 952 bp, which represents 88.32% of the entire genome. Additionally, we identified 72 tandem repeat sequences totaling 21,744 bp, which account for 0.48% of the coding gene length, and 74 dispersed repeat sequences totaling 5469 bp, constituting 0.12% of the coding gene length. A circular genome plot for the sample was generated using Circos software 0.69.6 to provide an overview of the distribution of genes, annotation information, GC content, and other genomic elements (Figure 5).

**Figure 6:**
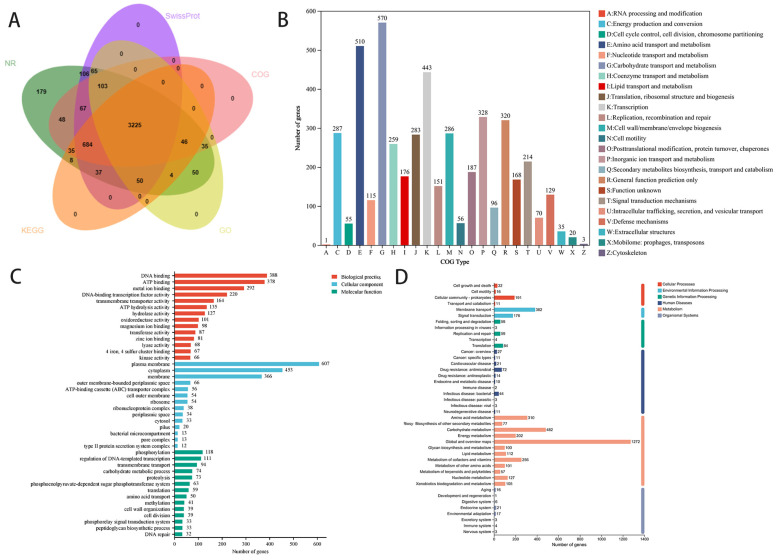
Gene annotation of YMK25. (**A**): Analysis of common and specific annotations in the basic database. (**B**): COG database annotation. (**C**): GO database annotation. (**D**): KEGG database annotation.

To further analyze the genomic information of strain YMK25, we compared its sequence data with the NR, SwissProt, COG, GO, and KEGG databases. A total of 4746 genes were annotated across these five databases. Specifically, the NR database annotated 4742 genes, SwissProt annotated 4337 genes, COG annotated 4243 genes, GO annotated 3578 genes, and KEGG annotated 4089 genes (Figure 6A).

The COG annotation results (Figure 6B) indicated that the identified genes were classified into 24 functional categories based on protein clustering into homologous groups. The major categories included carbohydrate transport and metabolism, amino acid transport and metabolism, transcription, inorganic ion transport and metabolism, with the top five functional categories containing 570, 510, 443, 328, and 320 genes, respectively, accounting for 51.16% of the annotated genes. This indicates that the strain has a stronger capacity and responsiveness for absorbing amino acids and carbohydrates from the environment.

The GO functional annotation results (Figure 6C) categorized the genes into three groups based on their sequence similarity to known sequences: Biological Process, Cellular Component, and Molecular Function. Among these categories, Molecular Function comprised the highest number of genes (3016), followed by Biological Process (2027) and Cellular Component (1773). Genes related to carbohydrate metabolism, flagellum-dependent cell motility, stress response, oxidoreductase activity, iron binding, and antioxidant activity are abundant in biological processes and molecular functions. These genes play a significant role in the rapid growth of the strain and in promoting plant growth.

KEGG database analysis revealed 5 primary categories and 42 secondary categories, with the highest number of annotated genes pertaining to metabolic functions (Figure 6D). In the tertiary classification, 227 pathways were annotated, including 8 pathways containing over 100 genes. Genes and pathways associated with plant growth promotion were identified, including amino acid metabolism, carbohydrate metabolism, and the metabolism of cofactors and vitamins. Overall, these genes support plant growth and metabolism by enhancing nutrient availability and providing resistance to biotic and abiotic stresses.

### 2.7. Prediction of Plant Growth-Promoting Genes in YMK25 Genome

The gene annotation results indicate that strain YMK25 contains diverse genes associated with plant growth and stress tolerance. These genes include those involved in siderophore biosynthesis, phosphatase activity, phosphate metabolism, auxin biosynthesis, and nitrogen fixation. Furthermore, the functions of these genes correspond with the functional characteristics validated in this study (Table 3).

Additionally, the strain also includes other plant growth-promoting related genes (*gcvA*, *pabB*, *cmoB*, *gshA*) and antibiotic synthesis related genes (*bpeT*, *cobS*). The annotation results identified genes associated with hydrolytic enzyme activities, including protease synthesis (*lepB*, *clpP*, *secA*), chitinase (*cpxA*, *cpxR*), and cellulase (*celA*, *celB*), as well as Na^+^/H^+^ antiporter proteins (*nhaA*, *nhaB*), suggesting potential salt tolerance. These findings suggest that strain YMK25 may possess the ability to inhibit the growth and reproduction of pathogenic microorganisms and to tolerate salt and alkaline conditions.

### 2.8. YMK25 Possesses a Growth-Promoting Effect on Maize Seedlings

**Figure 7:**
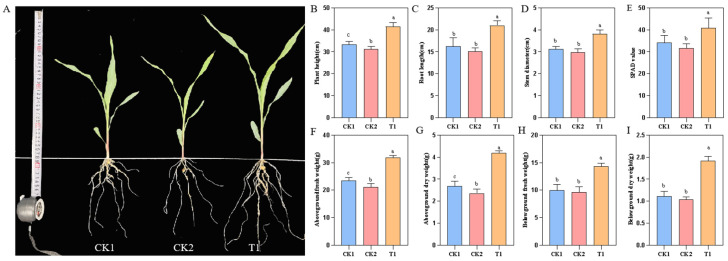
Evaluation of the promoting growth effect of strain YMK25 on maize seedlings. Note: (**A**): 14-day-old maize seedlings under different treatments; (**B**): plant height; (**C**): root length; (**D**): stem diameter; (**E**): SPAD; (**F**): aboveground fresh weight; (**G**): aboveground dry weight; (**H**): belowground fresh weight; (**I**): belowground dry weight. The different lowercase letters indicate significant differences among treatments (*p* < 0.05). The same below.

Pot experiments were conducted to investigate the promoting effect of the strains on maize growth, and the growth indicators of maize seedlings were measured after 14 d (Figure 7). The growth and physiological parameters of the treatment group increased to varying degrees. Maize seedlings in the T1 treatment group exhibited significantly greater plant height, root length, and stem diameter compared to the control groups (CK1 and CK2).

The chlorophyll content is critical for energy conversion and absorption. The SPAD values of maize leaves in the T1 treatment increased by 16.54% and 22.53%, compared to the control groups. This suggests that inoculation with strain YMK25 can enhance chlorophyll content in maize seedlings, likely due to the secretion of auxins by YMK25, which promotes overall plant growth and subsequently boosts chlorophyll synthesis. This indicates that YMK25 enhances root vitality by increasing the levels of chlorophyll, thereby promoting plant growth [19].

**Figure 8:**
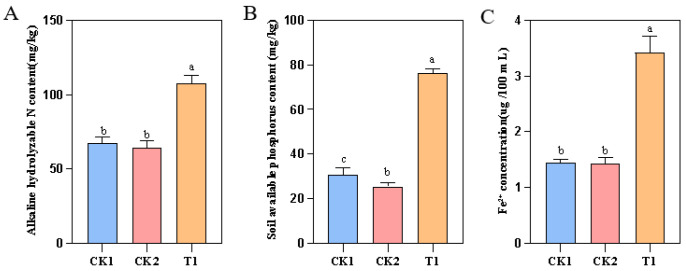
Effects of different treatments on soil nutrients. Note: (**A**): alkaline hydrolyzable N content; (**B**): available phosphorus content; (**C**): Fe^2+^ concentration.

Nitrogen (N) and phosphorus (P) are vital for soil nutrient cycling, ecological functions, and the growth and development of plants [20]. Compared to the control groups (CK1 and CK2), soil nutrient components in the T1 treatment exhibited significant improvements (Figure 8A,B), with alkaline hydrolyzable nitrogen and available phosphorus content increasing by 31.15% to 39.08% and 33.09% to 48.23%, respectively. This suggests that strain YMK25 has a phosphorus-dissolving effect, converting insoluble phosphorus in the soil into available phosphorus. This process not only compensates for phosphorus consumed by plant growth but also increases the content of readily available phosphorus. The concentration of ferrous ions in the soil after T1 treatment significantly increased by 31.15% to 39.08% (Figure 8C), suggesting that the addition of strain YMK25 enhances the concentration of Fe^2+^ available for plant uptake.

## 3. Discussion

The results of this study highlight the significant potential of strain YMK25 in enhancing maize growth through various mechanisms, suggesting its effective utilization in the development of microbial fertilizers that could positively impact both maize growth and soil nutrient levels.

### 3.1. In Vitro Evidence of YMK25’s Agricultural Potential

The in vitro experiments indicated that YMK25 can fix nitrogen, solubilize phosphorus, and produce both siderophores and IAA. Gao et al. [21] isolated six nitrogen-fixing strains from the rhizosphere soil of *Hippophae rhamnoides* L. Li et al. [22] reported that *Sphingobium* sp. SX14, isolated from the maize rhizosphere, demonstrated a capacity for inorganic phosphorus solubilization of 71.70 mg/L. *Burkholderia* sp. MEL01, isolated from rice-wheat rotation field soil, exhibited an organic phosphorus solubilization ability of 41.43 mg/L [23]. These functions are consistent with the findings of this study. Additionally, a previous study demonstrated that siderophores could function as biofertilizers and biopesticides by enhancing plant growth, nutrient uptake, and overall crop health [24]. IAA is a crucial plant growth regulator that influences various growth processes [25]. These abilities suggest that YMK25 may enhance the growth of maize seedlings.

### 3.2. Genomic Insights and Functional Annotation

Through comparative genomics, strain YMK25 was identified as *Klebsiella pneumoniae*. The genomic characterization of YMK25 revealed numerous genes associated with plant growth promotion and stress tolerance, including those involved in nitrogen fixation, phosphorus solubilization, and IAA biosynthesis. Similar genes were also predicted in the genomic analyses of plant rhizosphere-promoting bacteria [26,27]. Genome annotation findings indicate that YMK25 possesses a diverse array of functional genes that correspond with the strain’s phenotypic traits observed both in vitro and in pot experiments. These research findings enhance our understanding of the mechanisms through which YMK25 promotes plant growth and offer new directions for future applications in genetic engineering.

### 3.3. Application of YMK25 in Agriculture

PGPR plays an important role in enhancing crop vigor, yield, and stress resistance. They can serve as biostimulants to promote plant growth and improve quality and yield [28]. Pot experiments showed that strain YMK25 significantly improved maize growth, evidenced by increases in plant height, root length, stem diameter, and chlorophyll content. Additionally, inoculation with YMK25 had a positive impact on soil nutrient levels. Wang et al. [29] demonstrated that strain BW2-6, which has functions such as phosphorus solubilization and potassium dissolution, had the most significant effects on growth indicators, including plant height, stem diameter, and aboveground dry biomass of silage corn seedlings. Lv et al. [30] demonstrated that the application of functional microorganisms can enhance maize chlorophyll content. Li et al. [31] indicated that the strain *Peribacillus simplex* M1 possesses functions such as nitrogen fixation and IAA production. After inoculation, it increases chlorophyll content in maize leaves and promotes the growth of roots and leaves. Katsenios et al. [32] determined that *Bacillus mojavensis* can increase chlorophyll content in maize and enhance maize yield. Soil is an ecological niche for the presence of microorganisms, and inoculating beneficial microbes can promote soil revitalization. Research indicates that applying *Brevibacillus reuszeri* MPT17 treatment significantly increases the levels of available phosphorus and potassium in rhizosphere soil and the total potassium content in plant roots [33]. Li et al. [34] demonstrated that inoculating microorganisms can improve the content of soil nitrate nitrogen, total nitrogen, available phosphorus, and other nutrients. Zhang et al. [26] found that inoculation with *Leclercia adecarboxylata* LN01 can promote biomass accumulation in corn plants and effectively improve soil nutrient components, with total nitrogen and available phosphorus in the soil increasing by 31.15% and 33.09%, respectively. It is evident that although biostimulants do not classify as fertilizers, they can promote crop growth through various biochemical mechanisms due to the bioactive substances they contain. They can enhance soil nutrient utilization and promote plant growth and the absorption of mineral elements by activating chelates in the soil through their own metabolism, which includes the production of plant hormones, nitrogen fixation, phosphate solubilization, and the generation of iron carriers [3,35,36]. This is consistent with the results of this study. However, the interaction mechanism between the YMK25 strain and corn, its actual impact on corn yield, and its survival rate and persistence in field soils to enhance the application of these beneficial microorganisms in various agricultural systems will be key focus areas in our future research to provide more sustainable agricultural solutions.

## 4. Materials and Methods

### 4.1. Soil Samples and Strain Isolation

Soil samples were collected from the rhizosphere of healthy mature maize plants in the experimental field at BYAU Technology Park (125°21′ E, 46°24′ N) in September 2023, Heilongjiang, China. The experimental site is situated in a northern temperate continental monsoon climate zone characterized by an average annual rainfall of 427.5 mm, a frost-free period averaging 143 d, and an effective accumulated temperature of 2900 °C. The region’s soil is classified as calcareous black calcic soil. After uprooting the maize plants, soil particles adhering to the roots were carefully scraped. The collected soil samples were placed in sterilized, sealed bags and transported to the laboratory for further analysis.

Ten grams of rhizosphere soil and prepare 10^−4^ to 10^−6^ gradient dilutions using 1 L of sterile distilled water. From each soil dilution, inoculate 100 μL onto LB agar plates and incubate upside down at 30 °C for 3 d. Then, select colonies with distinct morphologies for isolation and purification until pure cultures are obtained.

Additionally, the collected soil samples were analyzed for their chemical and physical properties. The pH of the samples was measured as 8.54. The total salt content was found to be 1.37 g/kg. The levels of available nitrogen, phosphorus, and potassium were found to be 93.54 mg/kg, 56.78 mg/kg, and 212.7 mg/kg, respectively. The organic matter content of the soil was measured at 33.7 g/kg, and the soil moisture was 21.8%.

### 4.2. Assessments of Strain Promoting Properties In Vitro

The nitrogen-fixing function was determined by preparing a nitrogen-free Ashby medium according to the method described by Nautiyal [37]. The strains were incubated at 30 °C for 3 d, with the appearance of a transparent zone around the colonies as an indicator for identifying PGPR strains.

The phosphate-solubilizing ability of the bacteria was evaluated in NBRIP agar medium and PSM agar medium, following the methods described by Nautiyal [37] and Eida et al. [38]. The diameters of the transparent zone (D) and the colonies (d) were measured to provide a preliminary assessment of the strains’ ability to solubilize phosphate. The phosphate-solubilizing index (PSI) was calculated using the formula PSI = D/d [39]. The phosphorus-solubilizing ability was qualitatively evaluated according to the method described by Kumari et al. [40].

A colorimetric assay was used to assess the strains’ capacity to produce IAA [41]. The capacity for IAA production was determined based on the method described by Glickmann et al. [42]. The bacterial suspension was centrifuged, and the resulting supernatant was mixed with an equal volume of Salkowski reagent. The concentration of IAA was measured by spectrophotometry.

The capacity to produce a siderophore, an iron-chelating complex, was assessed using Chrome Azurol-S (CAS) agar medium as outlined by Yu et al. [43]. Quantitative assessments of siderophore production were conducted using the CAS assay, as previously described by Schwyn et al. [44] and Kang et al. [45]. Inoculate the bacterial suspension at 3% into the MKB liquid culture medium. After 48 h, centrifuge to collect the supernatant, and mix the supernatant with CAS detection solution. After reacting at room temperature for 1 h, measure the OD630 nm, designated as As. Meanwhile, measure the absorbance of the supernatant from the uninoculated MKB culture medium, designated as Ar. The relative expression of siderophores was calculated as follows:

Relative expression (%) = (Ar − As)/Ar × 100.

### 4.3. Determination of Basic Physicochemical Properties of the Strain

The isolated strain was cultured on LB medium at 30 °C for 24 h. The following characteristics were assessed: colony color, Gram reaction, gelatin hydrolysis, glucose fermentation, hydrogen sulfide (H_2_S) production, citrate utilization, methyl red reaction, and Voges–Proskauer (VP) reaction, among others. All analyses were performed according to the methods described by Holt et al. [46].

### 4.4. Identification and Genomic Analysis of the Strain

To better understand the genetic mechanisms by which rhizobacteria promote plant growth, the genome of the promising isolate was analyzed. The de novo whole genome shotgun sequencing was conducted by Shanghai Meiji Technology Pharmaceutical Co., Ltd, located in Shanghai, China. Initially, libraries with varying insert sizes were constructed. Next, a comprehensive whole-genome sequencing analysis was conducted using second-generation sequencing technology based on the Illumina HiSeq 2500 platform. The resulting high-throughput sequencing data were processed using SOAPdenovo2 software for assembly. This software performs k-mer correction and subsequently assembles the data to construct the complete genomic information of strain YMK25. Upload the assembled sequence information to NCBI to obtain accession numbers (SUB15115312). Following assembly, genomic analysis and functional annotation were conducted to elucidate the characteristics and potential functionalities of the strain.

The 16S rRNA gene was extracted from the genome of strain YMK25 using the EzBioCloud database (https://www.ezbiocloud.net/), accessed on 23 December 2024, and sequence alignment was performed against the database to identify the closest phylogenetic relationships [47]. A phylogenetic tree was constructed using MEGA 11.0 software with the maximum-likelihood (ML) method.

Comparative analysis of the 16S rRNA gene sequences identified closely related model strains. The complete genome sequences of these strains were obtained from the NCBI genome database (https://www.ncbi.nlm.nih.gov/genome/), accessed on 25 December 2024, for further comparison with the genome sequence of strain YMK25. Average nucleotide identity (ANI) is considered a robust, genome-based standard for determining the species identity of genetically related microorganisms [48]. The ANIm values were calculated using JSpeciesWS (https://jspecies.ribohost.com/jspeciesws/), accessed on 28 December 2024, and the DDH (DNA-DNA hybridization) value was determined using the Genome-to-Genome Distance Calculator 3.0 (https://ggdc.dsmz.de/ggdc.php), accessed on 29 December 2024.

### 4.5. Analysis of Strain-Induced Growth Promotion in Maize

The maize cultivar Zhengdan 958, a mid-early maturing hybrid variety, was developed by the Institute of Food Crops at the Henan Academy of Agricultural Sciences [49]. A bacterial suspension was prepared by inoculating strain YMK25 into sterile LB liquid medium and culturing at 30 °C, 160 rpm for 48 h, and then diluting with sterile water to a concentration of 10^8^ cfu/mL. Uniform, healthy maize seeds of similar size were selected and surface-sterilized with a 0.9% sodium hypochlorite solution. The seeds were rinsed with sterile water and subjected to germination experiments in a constant temperature incubator at 30 °C under dark conditions until the shoots reached a length of 0.5 cm. The pot experimental treatments included CK1 (soaked in aseptic LB culture medium), CK2 (soaked in sterile water), and T1 (soaked in 10^8^ cfu/mL bacterial suspension), with each treatment replicated four times. The germinated seeds were sown in square pots (14.5 cm × 13.3 cm × 11.3 cm) filled with sterilized soil (V soil:V sand = 1:1), with one seedling placed in each pot. Irrigate the plants with 50 mL of either water, sterile LB liquid medium, or YMK25 bacterial suspension once every 7 d.

The maize plants grew to the V4 stage 14 d after sowing. Uniform maize seedlings were selected to measure plant height, stem diameter, root length, and fresh weights of both above-ground and below-ground parts. Fresh samples were heated at 105 °C for 30 min and then dried at 80 °C until constant weight was achieved. Subsequently, above-ground and below-ground dry weights were measured. Chlorophyll values were measured using a SPAD502 chlorophyll meter. Collect rhizosphere soil samples to measure total nitrogen and available phosphorus. The determination methods are based on Bao [50]. The determination of Fe^2+^ was performed using the o-phenantroline colorimetric method [51].

### 4.6. Data Statistics and Analysis

Data analysis was conducted using Microsoft Excel 2016 for data organization and preliminary analysis. Significant differences were assessed using SPSS 26.0 software, applying the Least Significant Difference (LSD) method for statistical analysis (*p* < 0.05). SPSS was also used to perform correlation analysis. The results were visualized using GraphPad Prism 8.

## 5. Conclusions

This study successfully isolated a bacterial strain, designated YMK25, identified as *Klebsiella pneumoniae*, which exhibits significant growth-promoting effects. This strain possesses several beneficial traits, including nitrogen fixation, solubilization of inorganic and organic phosphorus, siderophore production, and the synthesis of indole-3-acetic acid. Whole-genome analysis revealed a substantial number of genes associated with these growth-promoting functions, providing valuable insights for future research. The application of strain YMK25 significantly enhances seedling growth, increases chlorophyll content, and improves soil nutrient levels. These findings suggest that strain YMK25 has considerable potential to promote maize development and to be utilized in formulating microbial fertilizers.

## Figures and Tables

**Table 1:** Physiological and biochemical characteristics of strain YMK25.

Identification Items	Strain YMK25	Identification Items	Strain YMK25
Methyl red reaction	−	Arginase utilization	−
Voges–Proskauer (VP)	+	Lysine decarboxylase utilization	+
Glucose fermentation test	+	Ornithine decarboxylase utilization	−
Lactose fermentation test	+	Gelatin hydrolysis	−
Citrate utilization	+	Hydrogen sulfide (H_2_S)	−
Malonate utilization	+	Urease utilization	+

**Table 2:** Genome features of YMK25 and comparison with other *Klebsiella* relatives.

Features	1	2	3	4	5
Total length of the genome (bp)	5,115,280	5,545,784	5,465,736	5,442,219	5,280,350
G+C content (%)	57.61	57	58	57.4	54.8
CDS (number)	4746	5162	5045	5037	4874
tRNA (number)	79	77	71	75	84

Note: 1: YMK25; 2: *Klebsiella pneumoniae* ATCC 13883; 3: *Klebsiella quasipneumoniae* 01A030; 4: *Klebsiella variicola* SB 5531; 5: *Klebsiella aerogenes* KCTC 2109.

**Table 3:** Predicting genes related to PGPR in the YMK25 genome.

PGPR Traits	Pathway
IAA	*trpA trpB trpCF trpE trpD trpR trpS trpGD ipdC patB aldh*
Siderophore	*tonB exbB entA entA entC entE fhuB fhuC fhuD fhuF efeB efeO efeU fepA fepB fepC fepG afuA afuB afuC sitB sitC sitD*
Nitrogen generation	*nirB nirD nasB nasC nifj nrtA nrtB nrtC ureA ureB ureC glnA glnB gltA gltB gltD narL narH*
Phosphate solubilization	*phoA phoB phoE phoR phoH pstA pstB pstC pstS phnA phnB phnC phnD phnE phnF phnG phnH phnI phnJ phnK phnM phnN*

## Data Availability

Data from this study are depicted in the figures and tables included in this article.
